# Revealing of Non-Cultivable Bacteria Associated with the Mycelium of Fungi in the Kerosene-Degrading Community Isolated from the Contaminated Jet Fuel

**DOI:** 10.3390/jof7010043

**Published:** 2021-01-11

**Authors:** Tatiana Shapiro, Konstantin Chekanov, Alina Alexandrova, Galina Dolnikova, Ekaterina Ivanova, Elena Lobakova

**Affiliations:** 1 Faculty of Biology, Lomonosov Moscow State University, 1-12 Leninskie Gory, 119192 Moscow, Russia; tatiananshapiro@gmail.com (T.S.); alina-alex2011@yandex.ru (A.A.); kachekanov@gmail.com (G.D.); elena.lobakova@gmail.com (E.L.); 2 Centre for Humanities Research and Technology, National Research Nuclear University MEPhI, 31 Kashirskoye highway, 115522 Moscow, Russia; 3 Department of General and Inorganic Chemistry, National University of Oil and Gas “Gubkin University”, 65 Leninsky Prospekt, 119991 Moscow, Russia; kostya_ne_kostya@mail.ru

**Keywords:** fuel biodamage, fuel biodegradation, microbial community, *Talaromyces*, *Penicillium*, *Aspergillus*

## Abstract

Fuel (especially kerosene) biodamage is a challenge for global industry. In aviation, where kerosene is a widely used type of fuel, its biodeterioration leads to significant damage. Six isolates of micromycetes from the TS-1 aviation kerosene samples were obtained. Their ability to grow on the fuel was studied, and the difference between biodegradation ability was shown. Micromycetes belonged to the *Talaromyces, Penicillium*, and *Aspergillus* genera. It was impossible to obtain bacterial isolates associated with their mycelium. However, 16S rRNA metabarcoding and microscopic observations revealed the presence of bacteria in the micromycete isolates. It seems to be that kerosene-degrading fungi were associated with uncultured bacteria. Proteobacteria, Actinobacteria, Bacteroidetes, and Firmicutes were abundant in the fungal cultures isolated from the TS-1 jet fuel samples. Most genera among these phyla are known as hydrocarbon degraders. Only bacteria-containing micromycete isolates were able to grow on the kerosene. Most likely, kerosene degradation mechanisms are based on synergism of bacteria and fungi.

## 1. Introduction

Fuel biodamage leads to a decrease in its quality, as well as corrosion of cutting and fuel tanks during storage and transportation [1,2,3,4,5,6,7]. Among a wide range of fuels, different types of gasolines are the most resistant to biodamage, whereas kerosene is the most vulnerable one [6,7,8,9,10]. However, the nature of higher vulnerability of kerosene is not elucidated in detail. In some works, it is speculated that overall hydrocarbon composition and total nitrogen content, as well as physicochemical parameters such as pH and temperature, are important factors for degradation of different types of fuel [7,8,10,11,12,13]. In aviation, where kerosene is a widely used type of fuel, biodamage leads to significant damage. Taking into account an annual growth of air transportation worldwide, this industry faces a serious costly challenge due to kerosene biodegradation [3,6,8]. Main features of kerosene contamination are the appearance of stable oil–water emulsions, acidification, and color and odor change [11].

The presence of water containing mineral salts is crucial for the development of biodamage [14,15]. In a dry fuel, microbial growth is not observed, but even a trace of water (0.01–0.02%) is sufficient for harmful microorganisms to grow [16]. Under industrial conditions, a fuel always contains water [15,16]. During fuel biodegradation, several processes take place: development of a water layer on the bottom of the tank containing wastes and microorganisms (i); development of biofilm on the surface of tanks (ii); and clogging of pipelines and filter systems of aircrafts (iii) [7,12,17]. Altogether it leads to a metal corrosion in the zone of the water layer and subsequent destruction or delimitation of protective coatings [7,9]. Thus, biodamage changes fuel chemical composition, on the one hand, and, on the other hand, it causes impurities of biological origin (e.g., fragments of microbial biofilms) [7,17,18].

Fuel biodamage is caused by bacteria, filamentous fungi, and yeast [6,8,10,12,19]. Filamentous micromycetes are the most dangerous among them because they form aggressive waste products and their mycelium can clog fuel systems [8,14]. The following fungal genera are common in biofuel: *Aspergillus, Penicillium, Fusarium, Amorphotheca, Neosartorya, Paecilomyces, Talaromyces, Graphium, Cladosporium, Candida, Yarrowia, Schizosaccharomyces, Saccharomyces*, and *Pichia* [3,7,8,11,13,19,20,21]. The main bacterial genera found in fuel are *Bacillus, Flavobacterium, Sarcina, Micrococcus, Rhodococcus, Pseudomonas, Comamonas, Burkholderia*, and *Klebsiella* [3,6,8,11,12,16,17,19,20,21]. The ability of microorganisms to biodegrade is due to their hydrolytic enzyme systems, and it is different depending on the substrates. The rate and severity of damage depends on fuel composition: linear hydrocarbons > branched hydrocarbons > aromatic hydrocarbons [10,11,12,20,22].

The majority of research on microbial biodegradation is focused on the pure cultures of bacterial or fungal strains isolated from the fuel [3,4,5,6,7,8,18,20,21,23,24,25,26]. However, in real systems, biodamage is caused by a complex impact of microbial communities [11,17,21,27,28,29]. Indeed, interactions of fungi and bacteria are common in a wide variety of habitats: decaying wood, human bodies, and water and soil environments [30]. Fuel-degrading micromycetes also exist in the form of the associations with bacteria. In this case, fuel degradation may be a synergetic process of microorganisms [5,6,31,32]. Most data on the coexistence of fungi and bacteria were obtained for soil systems. There bacteria interact with micromycetes forming close contact between each other [31,33]. On the one hand, bacteria provide fungal mycelium with more efficient settlement and substrate utilization [8,31]. They are related to a specific group of prokaryotes, hydrocarbon-oxidizing, or oleophilic bacteria, i.e., the bacteria that naturally utilize oils as their source of nutrients [8]. On the other hand, bacteria colonize a zone called the hyphosphere, which is formed around the fungal mycelium [32]. The hyphosphere is characterized by specific hydrostatic forces and the gradient of nutrients due to fungal exometabolites. In some cases, it was shown that the coexistence with fungal mycelium is necessary for biodegradation of hydrocarbons by the bacteria [34]. Fungi mediated attachment of nutrients particles (hydrocarbons) and bacteria to the network of the mycelium [32]. Fungi are responsible for transportation of nutrients [35,36,37] and bacterial cells [31,32,34] through the nexus of hyphae and provide a specific microenvironment for prokaryotes [32]. There are two types for the transport of organics and bacterial cells: “fungal highways”, through a thin layer of liquid environment with specific physicochemical parameters surrounding fungal hyphae, and “fungal pipelines”, or translocation inside hyphae [32,35,36,37]. “Fungal highways” promote the direct motility of bacteria in water-unsaturated environments, whereas “fungal pipelines” supply hydrocarbons to bacteria [32,35,36,37]. Furuno et al. [35] showed the possibility of using the mycelium of micromycetes for isolation of oil-degrading bacteria *Xanthomonas*, *Rhodococcus,* and *Pseudomonas* from the contaminated environment. Generally, co-interactions between fungi and bacteria enhance the bioremoval of organic pollutants [37]. In addition, a large number of exohydrolases secreted by micromycetes increase the bioavailability of hydrophobic organic pollutants in the environment and contribute to the effective biodegradation of hydrocarbons by bacteria [12,22,38,39].

So far, the data on the role of complex fungal and bacterial communities (but not their separate components) in fuel biodamage are scarce. Information about it may be essential for developing new strategies to prevent biodegradation. At the same time, the microbial communities from fuels may be considered as prospective agents for biodegradation of oil and fuel pollution. Application of the micromycete–bacterial communities during bioremediation of soils and waters contaminated with hydrophobic organic toxins is a promising area of research.

For the first time, to our knowledge, this study describes the community of micromycetes and uncultivated bacteria from the contaminated aviation kerosene (TS-1 jet fuel).

## 2. Materials and Methods

### 2.1. Sampling and Cultures Isolation

Micromycetes were isolated from two types TS-1 jet fuel samples of an aviation kerosene made according to the Russian standard (GOST 10227-86). One type was represented by colorless liquid; it had no features of contamination (pH 7.5). Another type exhibited signs of contamination, e.g., of microscopic flakes; they were also represented by colorless liquids (pH 7.5). Since fungi were isolated only from the samples with visible signs of contamination, the first type was considered as non-contaminated fuel. The samples were taken from the drain of jet fuel into steel barrels stored in a fuel and lubricants storage for 1.5 months. One sample was represented by colorless liquid; it had no features of contamination (pH 7.5).

Each sample of the TS-1 jet fuel was taken in 3 replicates represented by 250 µL of the fuel and was plated onto Petri dishes filled with selective organic media, for micromycetes isolation: the standard wort-agar medium (WA) [40] and Czapek medium (CM) [41]. After 3–10 days of incubation (23 °C), fungal colonies were transferred to a fresh medium, to obtain monosporal cultures, and incubated for 5 days. Monosporal fungal cultures were used for morphological identification and detection of growth capacity, with the presence of model mixture of hydrocarbons containing *n*-pentadecane, *n*-hexadecane, *n*-octadecane, and 1,2,4-trimethylbenzene (1.96% *v/v* of each compound).

In order to indicate the presence of cultured bacteria associated with the mycelium of the monospore fungal cultures, the following media were used: Rich medium [42] and Evans medium (EM) [43]. Previously, it was shown that EM and Rich medium are optimal for isolation of cultured heterotrophic bacteria from samples of contaminated oil and fuel [44,45,46]. All isolates of micromycetes were transferred either on liquid or solid media containing 2.0% (wt/*v*) agar. They were incubated for 10 days, at 26 °C.

### 2.2. Morphological Features of Micromycetes

Isolated micromycete cultures were subjected to microscopic analysis for their identification. The analysis of the micromycetes morphology was performed on the 7-day-old cultures grown on the CM with 2% (wt/v) yeast extract at 25 °C. Mycelium morphology was examined by light microscopy, on a microscope Leica DM 2500 (Leica Microsystems, Wetzlar, Germany) equipped with a DFC 7000T camera (Leica Microsystems, Wetzlar, Germany). Morphological features of fungal mycelium were evaluated by scanning electron microscopy (SEM). The samples were fixed with 2%(*v*/*v*) glutaraldehyde and dehydrated through graded ethanol series (including 100% ethanol) [47]. Then, they were transferred into absolute acetone, dried at the CO_2_ critical point on a Dryer HCP-2 (Hitachi, Tokyo, Japan), sputtered with gold and palladium in an IB-3 Ion Coater (Eico, Hitachinaka, Japan) [47], and examined under a scanning electron microscope JSM-6380LA (JEOL, Akishima, Japan).

The identification of the isolated micromycetes was done as per the guidelines [48,49,50,51,52,53,54,55] and general principles of fungal classification.

### 2.3. Growth Ability on the TS-1 Jet Fuel

Spore suspensions (volume of 0.3 mL; optical density at 540 nm was of 0.2) of the micromycetes were transferred into the 20 mL glass tubes containing 3 mL of the sterile TS-1 jet fuel and the EM medium. The tubes were incubated in a thermostat, at +28 °C, with shaking and monitored every 5 days for a month. The TS-1 jet fuel was sterilized by filtration through the 50 mm round nitrocellulose membrane filters with a pore diameter of 0.24 µm (Filter Solution, Moscow, Russia). Growth ability was scored visually by formation of mycelium flakes as: 0 points—no growth; 1 point—the appearance of medium turbidity, very small flakes; 2 points—flakes of medium size, easily distinguishable visually; 3 points—large flakes; 4 points—small clots; and 5 points—large mucus clots [56].

### 2.4. Express Assay for the Detection of 16S/18S rRNA Genes

To verify the presence of bacteria in the fungal cultures, an express method of 16S/18S rRNA detection was used. The genes of eukaryotic and prokaryotic small subunit rRNA were amplified by using the pair of universal primers (U515F: GTGYCAGCMGCCGCGGTAA and U1390R: TTGYACACACCGCCCGTC, designed in the study of Wang et al. [57]). The method is based on using primer pairs to highly conservative motives of small subunit rRNA. It is possible due to orthology between 16S rRNA and 18S rRNA genes [58]. With their help, it was possible to obtain amplicons of both prokaryotic and eukaryotic genes with the expected lengths of 900 and 1250 bp, respectively, for simultaneous express-detection of fungal and bacterial genes.

Genomic DNA was extracted from 100 mg of the mycelium with DNEasy PowerSoil Kit (Qiagen, Hilden, Germany) under sterile conditions. DNA was amplified on a Mastercycler Gradient DNA amplifier (Eppendorf, Hamburg, Germany), as described by Wang et al. [57].

### 2.5. Next-Generation 16S rRNA Amplicon Sequencing (16S rRNA Metabarcoding) and Bioinformatic Analysis

Genomic DNA extraction was performed from the 2 mL biomass samples of each micromycete, with a PowerSoil DNA Isolation Kit (MO BIO Laboratories, Inc., Carlsbad, CA, USA), according to the manufacturer’s protocol. The sequence corresponded to the hypervariable loop V4 of the 16S rRNA gene was amplified by polymerase chain reaction (PCR), using the primers pair F515: GTGCCAGCMGCCGCGGTAA and R806: GGACTACVSGGGTATCTAAT [59], fused with Illumina adapters, a pad, and a linker of two bases, along with barcodes. PCR amplification and sequencing libraries preparation were performed as described previously [60]. The libraries were sequenced on a MiSeq benchtop sequencer (Illumina, San Diego, USA), using a MiSeq 500 cycles kit (Illumina, San Diego, CA, USA) for 2 × 250 bp paired-ends sequencing.

Pretreatment of the datasets was done in QIIME v 1.9.1 [61]. To analyze next-generation 16S rRNA amplicon sequencing (16S rRNA metabarcoding) data, direct and reverse sequences were combined. The dataset was cleaned from adapter sequences, chimeric sequences, and sequences of unsatisfactory quality. Sequences less than 200 nt long and higher than 1000 nt long were removed. Homologs of mitochondrial sequences were searched by BLAST [62] in the NCBI GenBank. The analysis and data visualization were performed in VAMPS [63], using the reference 16S rRNA database Silva release 138 v. 1.9.5/1.4.3 [64]. Venn diagrams were generated by using an online tool (http://bioinformatics.psb.ugent.be/webtools/Venn/).

## 3. Results

### 3.1. Micromycetes Isolated from the TS-1 Jet Fuel

A total of six monospore micromycete cultures were isolated from the samples, with visible signs of contamination (Table 1 and Table 2). No fungal isolates were obtained from the sample without visual presence of contamination. Since fungi were isolated only from the samples with visible signs of contamination, the first type was considered as non-contaminated fuel.

**Figure 1:**
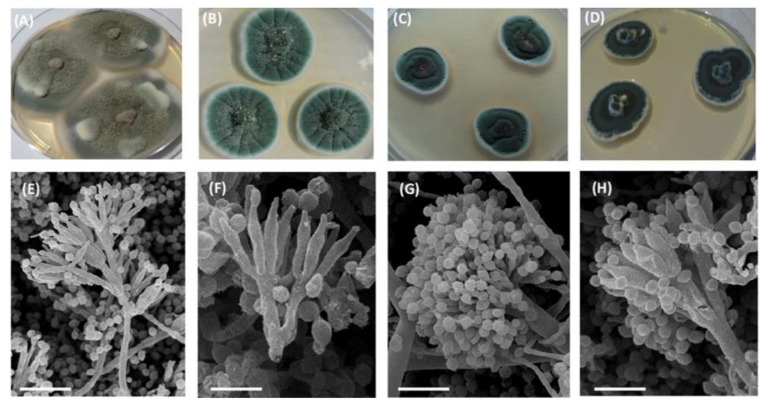
(**A**–**D**) Micromycete colonies on the solid culturing medium, seven days old. (**E**–**H**) Scanning electron images of micromycetes mycelium isolated from the TS-1 jet fuel. (**A**,**E**) *Talaromyces amestolkiae*, (**B**,**F**) *Penicillium chrysogenum*, (**C**,**G**) *Aspergillus sydowii*, and (**D**,**H**) *Talaromyces rugulosus.* Scale bar: (**F**) 5 μm, (**E**,**G**) 10 μm, and (**H**) 20 μm.

Fungal cultures were different in terms of colony type (Figure 1A–D) and sporulation morphology (Figure 1E–H). The cultures were different in terms of colony shape, color, sporulation, size, and morphology of conidia and conidiophores. The features of the obtained micromycete cultures are summarized in Table 1.

**Figure 2:**
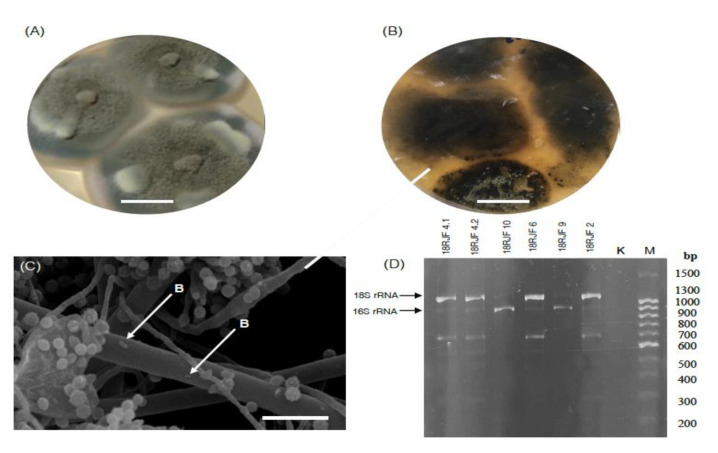
Analysis of the bacterial component of the communities isolated from the TS-1 jet fuel. (**A**) Native cultures of the micromycetes. Scale bar: 10 mm. (**B**) Mycelium lysis. Scale bar: 10 mm. (**C**) Bacteria attachment to the fungal hyphae in the isolate 18RJF9. Scale bar: 10 μm, arrows indicate the bacterial cells—B. (**D**) Separation of the PCR products obtained by using the primers for 16S rRNA detection in agarose gel. K is the negative control; M is the 100+ bp DNA ladder.

Collectively, based on morphological analysis and fungal colonies characteristics, the obtained micromycetes belonged to the Eurotiales ascomycetes: *Talaromyces amestolkiae* (Figure 1A,E), *Penicillium chrysogenum* (Figure 1B,F), *Aspergillus sydowii* (Figure 1C,G), and *T. rugulosus* (Figure 1D,H). At the initial stages of cultivation, fungal colonies were recognized as geometrically correct, without visible signs of damage (Figure 2A). At the same time, after storage of the micromycetes isolates for 30 days on Petri dishes, lysis of the micromycetes mycelium occurred (Table 2 and Figure 2B).

No single approach resulted in the growth of the cultured bacteria in all the fungal monospore isolates (data not shown). Although bacterial growth was detected neither at the stage of fungi isolation nor after mycelium lysis, SEM evidence revealed single bacterial cells associated with fungal hyphae (Figure 2C).

### 3.2. Bacterial Component of the Micromycetes’ Biomass Isolated from Jet Fuel Communities Revealed by 16S rRNA Data

The 16S/18S rRNA PCR analysis of the biomass of micromycetes revealed the presence of two amplicons (Figure 2D) with the length of 1250 bp (in 18RJF6, 18RJF4.2, and 18RJF2) and 900 bp (18RJF4.1, 18RJF4.2, 18RJF6, 18RJF9, and 18RJF10). The 900 bp fragment corresponded to bacterial 16S rRNA gene, whereas the 1250 bp fragment corresponded to eukaryotic 18S rRNA (obtained due to non-specific annealing of the primers with eukaryotic 18S rRNA). No 900 bp product was observed in the 18RJF2. Thus, the biomass of five of the six isolates of micromycetes isolated from the TS-1 jet fuel contained both bacteria and micromycetes and represented micromycete–bacterial communities. The presence of bacteria was also confirmed by SEM observations (Figure 2C). Single bacterial cells were observed on the mycelium in some cases.

**Figure 3:**
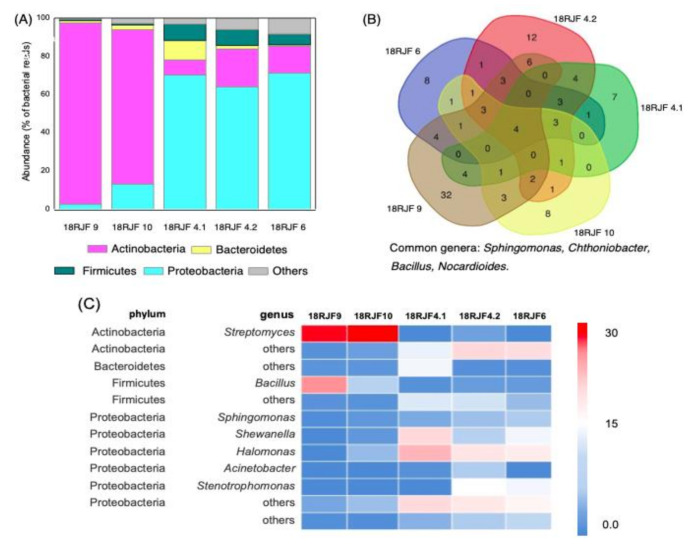
(**A**) Abundance of bacterial phyla in the communities isolated from the TS-1 jet fuel based on the sequencing of 16S rRNA libraries. The percentage of bacterial NGS reads corresponding to each phylum (of total prokaryotic reads) is shown. “Others” are unidentified sequences. (**B**) The number of common genera in the communities isolated from the TS-1 jet fuel based on 16S rRNA metabarcoding data analysis. (**C**) Frequency heatmap based on 16S rRNA sequencing. The color corresponds to the fraction of NGS reads in each isolate.

Most of the reads in the 16S rRNA metabarcoding datasets were represented by fungal mitochondrial 16S rRNA due to its orthology with bacterial 16S rRNA [58]. Based on our homologs search in the NCBI GenBank database, the highest homology of mitochondrial reads was observed with that of genera *Talaromyces*, *Penicillium*, and *Aspergillus* (98–100%, 99–100% coverage, Table 3). The sequences were deposited into NCBI GenBank, under the accession numbers listed in Table 3. Thus, the putative taxonomical affiliation with micromycetes obtained based on microscopic observations was confirmed by the 16S rRNA metabarcoding analysis. The number of bacterial genera in the isolates was in the range of 39–110. The percentage of unidentified reads after removing fungal sequences from consideration did not exceed 9%. The 16S rRNA-based metabarcoding study of the jet fuel community of micromycetes containing bacteria (18RJF4.1, 18RJF4.2, 18RJF6, 18RJF9, and 18RJF10) showed the absence of Archaea and presence of four predominant bacterial phyla, Proteobacteria, Actinobacteria, Firmicutes, and Bacteroidetes (Figure 3A). The vast majority of bacteria in all isolates was related to Gram-negative clades. The Isolate 18RJF4.2 had the highest prevalence of the phylum Bacteroidetes (0.4%). Actinobacteria were predominant in 18RJF9 and 18RJF10, with a relative abundance of 92% and 76%, respectively. In 18RJF4.1, the phylum Proteobacteria was present with a relative abundance of 99%. In three isolates (18RJF4.1, 18RJF4.2, 18RJF4.6), relatively high fraction of bacterial reads corresponded to Bacteroidetes were also detected (Figure 3A).

The communities studied contained four common bacterial genera: *Sphingomonas*, *Chthoniobacter*, *Bacillus*, *Nocardioides* (Figure 3). In 18RJF9 and 18RJF10, most of the reads corresponded to *Streptomyces*, 56% and 69%, respectively (Figure 3C); other Actinobacteria genera were not abundant. Despite the relatively high total number of Bacteroidetes, there were no prevalent genera related to this phylum (Figure 3C). In the 18RJF9, *Bacillus* (33%) was predominant. In the isolates 18RJF4.1, 18RJF4.2, and 18RJF6, the Proteobacteria *Shewanella* (6–18%), *Sphingomonas* (3–6%), and *Halomonas* (14–25%) were relatively abundant (Figure 3C).

### 3.3. The Growth Capacity of Isolated Communities on the TS-1 Jet Fuel

The growth capacity of the micromycete isolates on TS-1 fuel was assessed. The scores assigned to the isolates depended on the growth degree and varied from the absence of growth to the formation of large gray or light clots in the medium phase or on the phases’ boundary.

Communities derived from TS-1 jet fuel can be divided into 3 groups depending on their ability to decompose this fuel: (1) active destructors (five points), (2) potentially active destructors (four points), and (3) inactive or random (three or less points). The first group includes 2 isolates (18RJF4.2 and 18RJF6), the second—3 (18RJF4.1, 18RJF9 and 18RJF10), and the third—1 (18RJF1) (Table 2).

## 4. Discussion

Numerous studies in the last two decades have demonstrated that a wide variety of bacteria and fungi, including molds and yeasts, may colonize fuels such as diesel, biodiesel, and kerosene [7,8,11,15,65,66,67,68]. In addition, many of these microbes may actually degrade oil contaminations [3,4,5,11].

In five of the six isolates of micromycetes, we detected amplicones of bacterial 16S rRNA. Bacteria-containing isolates were passed through a pipeline of analysis of metabarcoding based on 16S rRNA gene. Since the mitochondrial gene of the ribosome small subunit RNA is an ortholog of the bacterial 16S rRNA gene, these genes are characterized by a relatively high degree of homology. Therefore, 16S rRNA libraries often contain mitochondrial reads [58]. In the isolates studied, a significant fraction of bacterial reads was represented by fungal sequences. Based on the homologs search, results of metabarcoding analysis revealed the presence of *Talaromyces*, *Penicillium*, and *Aspergillus* in the isolates. Although high homology of the mitochondrial ribosomal small subunit RNA gene is not solid evidence for precise micromycete identification, it is a confirmation of microscopic data and visual observations. Thus, three genera of micromycetes were isolated from the TS-1 jet fuel. Indeed, they are abundant in petroleum-contaminated soil and show kerosene-degrading activity [7,8,11,20,24,25,69]. *Penicillium* and *Aspergillus* are considered to be the most effective utilizers of hydrocarbons (particularly kerosene fuel) among fungi [24,25], but *Aspergillus* demonstrates a higher growth rate [24]. It should be noted that the same fungi were isolated from oil samples in different geographical regions and under different experimental conditions [2,3,4,5,6,8]. In this regard, they may be considered as widespread fungal components of such kerosene microbial communities.

In the isolates where 16S rRNA amplicons were detected, hyphae lysis of the micromycetes was observed after 30 days of cultivation. In fact, lysis of fungal mycelium is associated with the presence of vigorously dividing bacteria [70]. Moreover, single bacterial cells were observed on the surface of hyphae of some isolates. In our previous works, thirteen bacterial strains were isolated from the contaminated TS-1 aviation kerosene by conventional microbiological methods on the Rich medium. Their taxonomic affiliation was verified by 16S rRNA sequencing [45,46]. Kerosene was characterized by a diverse bacterial composition that included the following genera: *Sphingobacterium, Alcaligenes, Rhodococcus,* and *Deinococcus*. Isolated strains were capable of the degradation of hydrocarbons and production of biosurfactants [45,46]. At the same time, in this study, the same bacterial genera were indicated in monospore fungal cultures based on 16S rRNA metabarcoding. However, it was impossible to isolate bacteria from these communities from the TS-1 jet fuel grown on the Rich medium, WA, CM, and the EM after five days of incubation (see Materials and Methods). It might indicate a close relationship between fungi and bacteria.

Prokaryotes are known as effective hydrocarbon-utilizing microorganisms [12,19]. The most common fuel=degrading bacteria are related to Firmicutes, Bacteroidetes, Actinobacteria, and Proteobacteria [13,17,29]. A total of 16S rRNA metabarcoding data revealed the presence of these main phyla of oil-degrading bacteria in all the samples of TS-1 jet fuel. They were detected previously and isolated from oil-contaminated soils and samples of petroleum [12,39]. In addition, effective oil degradation has been reported for some archaea, e.g., Euryarchaeota, Thaumarchaeota, and Crenarchaeota. Oil-contaminated soils and ponds are characterized by very complex archaeal communities, and their taxonomic structure is more complex than that of bacteria [71,72]. Especially, halophilic archaea are abundant in habitats with high salinity [73]. At the same time, no significant number of archaea was detected in the studied TS-1 jet fuel sample.

Many bacterial genera with the ability to degrade hydrocarbons were isolated from soil or water bodies contaminated by oil. *Mycobacterium*, *Arthrobacter*, *Marinobacter*, *Achromobacter*, *Alcaligenes*, *Corynebacterium*, *Flavobacterium*, *Micrococcus*, *Nocardia*, *Pseudomonas*, *Bacillus, Dietzia, Gordonia, Halomonas, Cellulomonas, Rhodococcus*, and *Alcanivorax* are especially common petroleum degraders [8,13,17,26,74]. All studied communities contained bacterial genera with the reported ability to perform petroleum degradation (Table 3); thus, they might be involved into kerosene biodamage as well, e.g., *Sphingomonas*, *Bacillus*, *Rhodococcus*, *Nocardioides*, *Halomonas, Pseudomonas*, *Stenotrophomonas*, *Arthrobacter*, and *Streptomyces*. The Actinobacteria *Streptomyces* was predominant in two TS-1 samples. It is able to degrade linear hydrocarbon and polycyclic aromatic hydrocarbons, as well as hydroxyalkanoates [8,12,17,26,39,75]. In three isolates, a relatively high amount of *Halomonas*, *Shewanella*, *Stenotrophomonas*, and *Acinetobacter* was observed, and, in one isolate, *Bacillus* was predominant. Petroleum degradation is well-known for *Shewanella*, *Stenotrophomonas*, and *Acinetobacter* [12,19,67,76]. Growth and degradation of hydrocarbons by *Halomonas* have been described under hypersaline conditions and in marine environments [74,77]. Some of these bacteria show biosurfactant-producing activity, which is important for effective utilization of hydrophobic substrates [12,76]. Notably, *Bacillus*, *Nocardioides*, and *Sphingomonas* were observed in all isolates studied.

All studied isolates, namely 18RJF6, 18RJF4.2, 18RJF9, 18RJF10, and 18RJF4.1—where the first two belonged to the group of active hydrocarbon degraders and the remaining potentially active destructors—contained bacterial component in their hyphosphere. Based on our results, there was no correlation between micromycete genus and TS-1 jet fuel biodegradation. Since these isolates exhibited different bacterial composition, it raises the question of whether qualitative and quantitative characteristics of bacterial components of such communities determine effectiveness of fuel degradation. This sparks a scientific quest to reveal the numerical criteria of “successful” fuel-degrading community. For this purpose, it will be necessary to collect a high number of new data of such communities isolated from fuel, to subject them to statistical analysis of generated metabarcoding data. Nevertheless, we took an attempt to find some common patterns of active fuel-degrading communities based on our primary data. First, active fuel-degrading communities were characterized by high total diversity of Actinobacteria, whereas potentially active (with an exception of 18RJF4.1) were characterized only by a high abundance of *Streptomyces* and low number of others (Figure 3C). Second, active and potentially active communities contained high numbers of reads of either *Bacillus* or *Halomonas* and *Stenotrophomonas*, which are fuel-degrading bacteria (Figure 3C). In general, active and potentially active communities contained a higher number of abundant bacterial genera with reported fuel degradation. When growing in fuel, hydrocarbon-oxidizing bacteria form communities that make up a single chain of hydrocarbon oxidation [8,17]. Each microorganism in such a community uses predominantly certain groups of hydrocarbons along specific metabolic pathways. When communities of microorganisms are exposed together, both a larger amount and a wider range of hydrocarbons are extracted from oil and petroleum products. One can speculate that abundance of more than one (and better several) genera of fuel-destructing bacteria is important for “successful” fuel degradation by a community. It can be explained by higher diversity of genus-specific enzymes degrading potentially higher numbers of hydrocarbons.

The existence of petroleum-degrading microorganisms in the community raises the question of their synergism in substrate utilization and biodamage of the fuel. The presence of uncultivated forms of bacteria might indicate their very close interactions with fungi. Formation of a stable community between bacteria and fungal mycelium is a strategy for their coexistence [28,33,78,79]. Synergism of bacteria and fungi in substrate utilization is a well-known phenomenon. Bacteria exhibit a diverse enzymatic system for catabolism of aromatic and aliphatic, saturated and desaturated, and branched and unbranched hydrocarbons [7,8,12,80]. They use a broad range of oxygenases to insert oxygen atoms to organic molecules [8,81]. The fungal genome also encodes diverse enzymes that transform a wide range of hydrocarbons, which are expressed to utilize such substrates [8,12,27,82]. In contrast to bacteria, most fungi express extracellular oxidoreductases acting beyond the zone of microorganisms’ growth [32,83]. It makes it possible to oxidize extremely hydrophobic substrates to their more hydrophilic derivatives, which may diffuse to fungi and bacteria [83]. Thus, the communities of fungi and bacteria implement co-metabolism of fuel: It was shown that these components cannot degrade some hydrocarbons separately [83]. Fungi provide a microenvironment favorable for bacteria in their hyphosphere. It is essential for the establishment constant metabolic crosstalk, as well as immobilization of motile bacteria walking in the environment [32]. In our work, the presence of bacteria on the mycelium is indirect evidence of such interactions. The network of mycelium serves as a “transport vector” for dispersal movement of bacteria by “fungal highways” and for transport of hydrocarbons and metabolites by “fungal pipeline” to bacterial cells [28,31,32,34,35,36,83]. Moreover, some fungi form endosymbiosis with bacteria; therefore, they may also be transferred by the “pipeline” [33,78]. Communities of hydrocarbon-degrading fungi and bacteria form biofilms [17,18,28,83,84]. Genes for biofilm formation were detected in the fungal-interactive bacterium *Burkholderia terrae* BS001 [80]. Hydrocarbon-degrading bacteria live in a hydrophilic environment. At the same time, their immobilization in the vicinity of a substrate is essential for degradation [28,46,83]. Thus, biofilm formation is important for microorganisms anchoring on a hydrophobic surface [83]. Extracellular polymeric substances are an important mandatory part of all biofilms [85]. They form a network of extracellular matrixes that accumulates hydrocarbons and exhibits sites of hydrophobic-substance binding [83]. Thus, biofilm formation increases hydrocarbon availability to microorganisms. Moreover, biofilm formation increases stability of the microbial community under adverse conditions [84,85]. Additionally, microorganisms may be involved in the formation of multispecies biofilms, providing stability for formed communities [85]. Numerous recent studies [84,86,87] have shown a promising trend in the applications of the fungal–bacterial biofilm in diverse fields.

Collectively, according to the literature sources, communities between hydrocarbon-degrading fungi and bacteria seem to be a form of microbial associations. They have greater degradable potential than pure cultures. In the current research, we, at the first time, isolated micromycete cultures from the TS-1 jet fuel. The fruits of the work already include a confirmation of an importance of community formation for better hydrocarbon utilization and high degree of interaction between oil-degrading fungi and high degree of interaction between fungi and bacteria in such communities. The inability to isolate bacteria associated with fungal mycelium and fuel degradation only by bacteria containing mycelium gives the task ahead to evaluate the presence of bacteria in fungal isolates from different types of fungi. It may be important for development of new strategies for prevention of fuel biodamage and hydrocarbon pollutants’ biodeterioration.

## 5. Conclusions

Micromycetes and bacteria are called biodegraders of fuel, including TC-1 jet fuel. In this paper, we studied the 16S rRNA metabarcoding of the biomass of micromycetes isolated from TS-1 jet fuel and showed that they are mainly micromycete bacterial communities with non-cultured bacterial forms. The high efficiency of hydrocarbon-oxidizing bacteria and fungi in the destruction of hydrocarbons is widely known. However, according to our data, only isolates of micromycetes that are communities with bacteria are effective in terms of degradation of TS-1 jet fuel, whereas the isolate of a micromycete devoid of bacteria is not capable of growth on fuels. These data are valuable for developing new strategies for protecting fuels from biodegradation and creating new approaches to waste biodegradation.

## Figures and Tables

**Table 1:** Cultural and morphological characteristic of isolated micromycetes.

Isolate	Cultural Characteristics	Morphological Characteristics	
		Mycelial Morphology	Sporulation Characteristics
18RJF6	Widely grown, velvety surface with individual	1–2 mm height	Sporulation: grayish-green, in some cases, the edge of the colonies lighter
			Conidiophores: 100–150 μm × 2.5–3 μm Typically, biverticillate and symmetrical; each conidiophore contained 3–5 metules and lanceolate phialides
18RJF9	felted areas; smooth reverse side; with exudate; reddish brown		
18RJF10			Elliptic conidia: 2–3 µm × 1.5–2.5 μm; smooth or slightly rough
18RJF2	Colonies of 10–20 mm in diameter with low growth; velvety surface, rugose	1–2 mm height	Sporulation: bright green
			Conidiophores: 40–110 μm × 2–3 μm
			Typically, biverticillate and symmetrical, as a rule, with additional branches (10–25 μm length); each conidiophore contained 5 or 6 metals and were from lanceolate to flask-shaped phialides
			Elliptic to fusiform conidia: 2.5–6 μm × 2.5–4 μm; smooth or slightly rough
18RJF4.1	Colonies of 2.5–3.5 mm in diameter; moderate growth rate; velvety surface; radially folded; colorless or yellow drops of exudate	1–3 mm height	Sporulation: yellow-green
			Conidiophores: 250–500 μm × 2.5–3.5 μm
			Typically, terverticillate and asymmetric, with a pressed lateral twig; each conidiophore contained 5 or 6 metules and from 3 to 6 bottle-shaped phialides
			Subspherical to ellipsoidal conidia: 3.0–4.0 μm × 2.8–3.8 μm; smooth
18RJF4.2	Colonies of 10–20 mm in diameter with slow growth rate; strongly folded with a well-defined edge;	2–4 mm height	Sporulation: blue-green
			Conidiophores formed on the substrate mycelium only: 500 μm × 5–8 μm
	the reverse side radially folded, initially without a specific coloration and then wine-purple		The spore heads biseriate radial, up to 20 µm in diameter; metules (6–7 μm length), phialides 7–10 µm × 2.0–2.5 µm
			Globular conidia: 2.5 µm × 3.5 µm; prickly, green in mass

**Table 2:** Characteristics of micromycetes isolated from the TS-1 jet fuel: sample isolation medium, micromycete species based on microscopic observation. degradation score of the TS-1 jet fuel scored as: 0 points—no growth; 1 point—cloudy solution, very small flakes; 2 points—flakes of medium size, easily distinguishable visually; 3 points—large flakes; 4 points—small clots; and 5 points—large clots. Mycelium lysis in the culture and visual presence of bacteria in the mycelium. EM—Evans medium, CP—Czapek medium, and WA—worth agar.

Isolate	Isolation Medium	Micromycete	TS-1 Degradation Score	Mycelium Lysis
18RJF2	EM	*Talaromyces rugulosus*	0	−
18RJF4.1	CP	*Penicillium chrysogenum*	3	+
18RJF4.2	CP	*Aspergillus sydowii*	5	+
18RJF6	WA	*Talaromyces amestolkiae*	5	+
18RJF9	WA	*Talaromyces amestolkiae*	3	+
18RJF10	WA	*Talaromyces amestolkiae*	2	+

**Table 3:** Microorganisms in the samples of the TS-1 fuel based on rRNA sequencing data: fungal genera based on mitochondrial DNA sequence, as well as their NCBI GenBank IDs and bacteria with putative oil degradation activity.

Isolate	Mitochondrial rRNA	GenBank ID	Bacteria with Putative Petroleum Destruction Activity
18RJF2	*Talaromyces*	MW393516	*None*
18RJF4.1	*Penicillium*	MW393517	*Sphingomonas*, *Bacillus*, *Rhodococcus*, *Halomonas*, *Nocardioides*
18RJF4.2	*Aspergillus*	MW393518	*Sphingomonas*, *Bacillus*, *Pseudomonas*, *Stenotrophomonas*, *Arthrobacter*, *Halomonas*, *Nocardioides*
18RJF6	*Talaromyces*	MW393519	*Sphingomonas*, *Bacillus*, *Pseudomonas*, *Stenotrophomonas*, *Arthrobacter*, *Halomonas*, *Nocardioides*
18RJF9	*Talaromyces*	MW393520	*Sphingomonas*, *Bacillus*, *Pseudomonas*, *Stenotrophomonas, Arthrobacter*, *Streptomyces*, *Nocardioides*
18RJF10	*Talaromyces*	MW393521	*Sphingomonas*, *Bacillus*, *Arthrobacter*, *Halomonas*, *Streptomyces*, *Nocardioides*

## Data Availability

The data presented in this study are openly available in GenBank at reference numbers: MW393516, MW393517, MW393518, MW393519, MW393520, MW393521.
